# Inhibition of the Prefrontal Projection to the Nucleus Accumbens Enhances Pain Sensitivity and Affect

**DOI:** 10.3389/fncel.2018.00240

**Published:** 2018-08-13

**Authors:** Haocheng Zhou, Erik Martinez, Harvey H. Lin, Runtao Yang, Jahrane Antonio Dale, Kevin Liu, Dong Huang, Jing Wang

**Affiliations:** ^1^Department of Pain, The Third Xiangya Hospital and Institute of Pain Medicine, Central South University, Changsha, China; ^2^Department of Anesthesiology, Perioperative Care and Pain Medicine, Langone Medical Center, School of Medicine, New York University, New York, NY, United States; ^3^Department of Neuroscience and Physiology, School of Medicine, New York University, New York, NY, United States

**Keywords:** corticostriatal circuits, prefrontal cortex (PFC), prelimbic cortex, nucleus accumbens (NAc), acute pain, chronic pain

## Abstract

Cortical mechanisms that regulate acute or chronic pain remain poorly understood. The prefrontal cortex (PFC) exerts crucial control of sensory and affective behaviors. Recent studies show that activation of the projections from the PFC to the nucleus accumbens (NAc), an important pathway in the brain’s reward circuitry, can produce inhibition of both sensory and affective components of pain. However, it is unclear whether this circuit is endogenously engaged in pain regulation. To answer this question, we disrupted this circuit using an optogenetic strategy. We expressed halorhodopsin in pyramidal neurons from the PFC, and then selectively inhibited the axonal projection from these neurons to neurons in the NAc core. Our results reveal that inhibition of the PFC or its projection to the NAc, heightens both sensory and affective symptoms of acute pain in naïve rats. Inhibition of this corticostriatal pathway also increased nociceptive sensitivity and the aversive response in a chronic neuropathic pain model. Finally, corticostriatal inhibition resulted in a similar aversive phenotype as chronic pain. These results strongly suggest that the projection from the PFC to the NAc plays an important role in endogenous pain regulation, and its impairment contributes to the pathology of chronic pain.

## Introduction

Pain protects us from injury and harm. In some cases, however, pain can become magnified, giving rise to exaggerated emotional responses (Melzack and Casey, 1968). In addition, acute pain can progress to chronic pain, resulting in long-term physical and psychiatric disabilities (Scudds et al., 1987; Petzke et al., 2003; Kehlet et al., 2006; Kudel et al., 2007; Scott et al., 2010). Research over the last 50 years has focused on understanding the pathologic mechanisms of nociception, particularly in chronic pain conditions, with the goal of finding interventions to repair maladaptive mechanisms. Few studies, however, have examined the ability of our central nervous system, especially the cortex, to regulate pain endogenously and show how such regulatory functions can be enhanced to treat pain.

The prefrontal cortex (PFC) is a highly evolved structure that projects to other cortical and subcortical regions to regulate a host of sensory and affective processes (Ressler and Mayberg, 2007; Fuster, 2009; Arnsten et al., 2012). Exogenous activation of the PFC has long been known to inhibit nociceptive withdrawal responses (Cooper, 1975; Hardy, 1985). Recently, optogenetic activation of the prelimbic region of the PFC (PL-PFC) in rodents has been shown to reduce not only nociceptive withdrawals, but also affective or aversive responses to pain (Lee et al., 2015; Zhang et al., 2015; Martinez et al., 2017).

At the circuit level, neurons in the PFC have been shown to project to the periaqueductal gray (PAG), which provides outputs to the rostral ventral medulla (RVM) to form a classic descending inhibitory circuit (Fields et al., 1983; Morgan et al., 1989, 1991; Ossipov et al., 2014). In addition, the PFC projects to the nucleus accumbens (NAc), a central node in the brain’s reward circuitry, which is known to play a role in pain regulation (Gear et al., 1999; Becerra et al., 2001; Magnusson and Martin, 2002; Becerra and Borsook, 2008; Geha et al., 2008; Gear and Levine, 2009; Baliki et al., 2010; Goffer et al., 2013; Navratilova and Porreca, 2014). Recent studies demonstrated that supraphysiologic activation of this PFC-NAc projection can alter pain phenotypes (Lee et al., 2015; Martinez et al., 2017). However, there has been no convincing evidence to demonstrate that in awake, free-moving animals, prefrontal neurons project to the NAc to inhibit pain. This is an important question, as chronic pain is known to alter the connectivity between these two regions (Apkarian et al., 2004; Baliki et al., 2012).

In the current study, we investigated the role of the PFC-NAc projection in the endogenous regulation of acute and chronic pain. We inhibited the pyramidal neurons of the PL-PFC using an optogenetic approach, and then measured pain behaviors using Hargreaves’ test, mechanical or cold allodynia, and conditioned place aversion tests. Next, we selectively inhibited the projection from the PFC to the NAc core. Our results showed that inhibition of this corticostriatal pathway profoundly enhanced both sensory and affective pain responses. This was the case in both naïve rats, as well as rats in the spared nerve injury (SNI) model of chronic neuropathic pain. Furthermore, the inactivation of this circuit in naïve rats reproduced the affective symptoms of chronic pain. Therefore, the projection from the PFC to the NAc likely plays a key role in the endogenous regulation of pain, and thus forms a target for therapeutic approaches.

## Materials and Methods

### Animals

All animal care and experimental procedures were approved by the School of Medicine, New York University, Institutional Animal Care and Use Committee (IACUC) and were consistent with the National Institute of Health (NIH) *Guide for the Care and Use of Laboratory Animals* (publication number 85-23) to ensure minimal animal use and discomfort. Male Sprague-Dawley rats were purchased from Taconic Farms, Albany, NY, USA and kept at Mispro Biotech Services Facility in the Alexandria Center for Life Science, with controlled humidity, room temperature, and 12-h (6:30 AM to 6:30 PM) light-dark cycle. Food and water were available *ad libitum*. Animals arrived to the animal facility at 250 g and were given on average 14 days to adjust to the new environment prior to the onset of experiments.

### Virus Construction and Packaging

Recombinant AAV vectors were serotyped with AAV1 coat proteins and packaged by the viral vector core at the University of Pennsylvania, Philadelphia, PA, USA. Viral titers were 5 × 10^12^ particles/mL for AAV1.CAMKII. NpHR-eYFP.WPRE.hGH and AAV1.CAMKII.eYFP.WPRE.hGH.

### Stereotaxic Intracranial Viral Injections and Optic Fiber Implantation

As previously described (Goffer et al., 2013; Lee et al., 2015), male rats were anesthetized with Isoflurane (1.5%–2%). Virus as specified above was delivered to the PL-PFC. Rats were bilaterally injected, using a 32 gauge 1 μL Hamilton syringe, with 0.6 μL of AAV1.CAMKII.NpHR-eYFP.WPRE.hGH or AAV1.CAMKII.eYFP.WPRE.hGH slowly at AP: +2.9 mm; ML: ±1.6 mm; DV: −3.7 mm with tips angled 12.5° toward the midline. The microinjection needles were then left in place for an additional 10 min, so as to allow for diffusion of virus particles away from the injection site and to minimize the spread of viral particles along the injection tract. Rats were then implanted with 200 μm optic fibers and 2.5 mm ferrules (Thorlabs) in the PL-PFC with the following coordinates: AP +2.9 mm, ML ±1.6 mm, DV −2.7 mm with tips angled 12.5° toward the midline. For bilateral fiber implants in the NAc core, the following coordinates were used: AP +2.2 mm, ML ±2.8 mm, DV −5.7 mm with tips angled 12° toward the midline. Optic fibers were held in place by dental acrylic.

Following animal sacrifice, frozen brain sections were collected at a thickness of 20 μm using a Microm HM525 Cryostat. The sections were then and analyzed for viral expression and optic fiber localization with histological staining. Animals with improper fiber placement, or viral expression outside the PL-PFC were excluded from further analysis.

### Immunohistochemistry

Rats were deeply anesthetized with Isoflurane and transcardially perfused with ice-cold PBS followed by 4% paraformaldehyde (PFA) in PBS. Brains were fixed in PFA overnight and then transferred to 30% sucrose in PBS to equilibrate for 3 days as described previously (Lee et al., 2015). Twenty micrometer coronal sections were made with a cryostat and washed with PBS for 10 min. The sections were washed in PBS and coverslipped with Vectashield mounting medium. The sections were also made after viral transfer for opsin verification, and were stained with anti-rabbit GFP (1:500, Abcam, Cambridge, MA, USA #AB290), NeuN (1:200, Vector Laboratories, Burlingame, CA, USA), DAPI (Vector Laboratories, Burlingame, CA, USA), and CaMKII-α (6G9) mouse mAb (1:200, Cell Signaling Technology, Danvers, MA, USA, #50049) antibodies. Secondary antibodies were anti-rabbit IgG conjugated to AlexaFluor 488, and anti-mouse IgG conjugated to AlexaFluor 647 (1:500, Life Technologies, Carlsbad, CA, USA). Images were acquired with a Zeiss LSM 700 Confocal Microscope (Carl Zeiss, Thornwood, NY, USA).

### Spared Nerve Injury (SNI) Surgery

The SNI procedure was performed as described previously (Wang et al., 2011; Goffer et al., 2013; Lee et al., 2015). Briefly, after rats were anesthetized with isoflurane (1.5%–2%), the skin on the lateral surface of the left thigh of the rat was incised. A section was then made through the biceps femoris muscle to expose the sciatic nerve and its three terminal branches: sural, common peroneal and tibial nerves. The common peroneal and tibial nerves were tied with nonabsorbent 5-0 silk sutures at the point of trifurcation. The nerves were then cut distal to each knot, and approximately 5 mm of the distal ends were removed to prevent reattachment. Nerves were dissected but not cut in sham surgeries (control group). The muscle layer was then sutured closed, and the skin was stapled. Staples were removed before any behavioral experiments.

### Animal Behavioral Tests

Behavioral tests with optogenetic stimulation in the PFC were done 2–4 weeks after viral injection. Tests with stimulation in the NAc core were done approximately 6 weeks after injection to ensure optimal expression of opsins. Prior to behavioral tests, optic fibers were connected to a 589 nm laser diode through an FC/PC adapter (Shanghai Dream Lasers, Shanghai), and laser intensity was measured with a power meter (Thorlabs, Newton, NJ, USA). The laser was delivered using a TTL pulse-generating box (Tucker-Davis Technologies, Alachua, FL, USA).

#### Mechanical Allodynia Test

A traditional Dixon up-down method with von Frey filaments of logarithmically incremental stiffness (0.45, 0.75, 1.20, 2.55, 4.40, 6.10, 10.50, 15.10 g) was used to measure mechanical hypersensitivity as described previously (Chaplan et al., 1994; Bourquin et al., 2006; Wang et al., 2011; Su et al., 2016). Rats were individually placed in plexiglass chambers over a mesh table and acclimated for 30 min. Fifty percent withdrawal thresholds were calculated as described previously (Wang et al., 2011). Von Frey filaments were applied to the lateral one-third of right paws (in the distribution of the sural nerve) of rats as described previously (Brennan et al., 1996; Su et al., 2015).

#### Hargreaves’ Test (Plantar Test)

The Hargreaves’ test was performed to evaluate the response to acute thermal stimulation (Tawfic et al., 2014). We used a mobile radiant heat-emitting device with an aperture of 10 mm in diameter (37370-Plantar Test, Ugo Basile, Italy) to produce acute noxious thermal stimuli and measured the latency to paw withdrawal. Rats were placed individually in a clear plastic chamber and left to acclimate prior to testing. The mobile heat generator was aimed at the plantar surface of the rat’s hind paw, and an infrared intensity of 40 was used. The latency to paw withdrawal was recorded automatically. Paw withdrawals due to locomotion or weight shifting were not counted and the trials were repeated. Measurements were repeated five times at 5 min intervals on the right paw. Separate trials were conducted for baseline (no activation) and optogenetic activation. The averages of the five measurements for each trial were taken and analyzed.

#### Conditioned Place Preference (CPP)

Conditioned Place Preference (CPP) experiments were conducted in a standard three-compartment apparatus (Stoelting Co., Wood Dale, IL, USA) consisting of two large compartments of equal size joined by a tunnel (Lee et al., 2015). Rat movements were recorded by a camera and analyzed with ANY-maze software. The CPP protocol was modified from King et al. (2009), and it included preconditioning, conditioning, and testing phases. Preconditioning was performed across 3 days for SNI-treated rats. During preconditioning, animals were exposed to the environment with full access to all chambers for 30 min each day. On day 3, the movement of each rat was recorded for 15 min and analyzed to verify the absence of any preconditioning chamber preference. Animals spending more than an 80% (time spent >720 s) or less than 20% (time spent <180 s) of the total time in any chamber were eliminated from further testing or analysis (approximately 15% of total animals), as described in previous studies (King et al., 2009; De Felice et al., 2013; Lee et al., 2015). Following the preconditioning phase, the rats underwent conditioning for 4 days with alternative treatment-chamber pairings in the morning and afternoon. Half of the rats received light treatment-chamber pairing in the morning and no light treatment-chamber pairing in the afternoon. Half of the rats received no light (laser) treatment-chamber pairing in the morning and light treatment-chamber pairing in the afternoon. The other half of the rats received opposite treatments. Both light vs. no light treatments and chamber pairings were counterbalanced and at least 4 h separated morning and afternoon sessions. Rats were placed in one chamber without access to the other chamber, in the presence or absence of optogenetic treatment for 30 min during the conditioning period. On the testing day, the animals were placed into the neutral (conduit) chamber and had access to all chambers for a total of 15 min without any light treatment. During the testing phase, the movements of animals in each chamber were recorded, and the time spent in each chamber was analyzed by the ANY-maze software. Increased time spent in one chamber paired with or without light treatment indicated preference for the chamber.

For acute pain CPA on naïve rats, we modified the classic CPA with a single day protocol (Johansen et al., 2001; Johansen and Fields, 2004; King et al., 2009; De Felice et al., 2013; Lee et al., 2015). This modified CPA protocol included preconditioning (baseline), conditioning and testing phases (10 min during each phase). Conditioning boxes were positioned on top of a metal mesh stand during all three phases (preconditioning, conditioning and testing). The phases occurred one immediately after the other. Animals spending more than 80% or less than 20% of the total time in either chamber in the preconditioning phase were eliminated from further analysis (approximately 20% of total animals). Immediately following the pre-conditioning phase, the rats underwent conditioning for 10 min. In both chambers, rats received a pin prick (PP) to a hind paw with a 27 g needle. This noxious mechanical stimulus was repeated every 10 s. Optogenetic activation was paired with one of the treatment chambers, and no light treatment was paired with the opposite chamber. Optogenetic stimulation and chamber pairings were counterbalanced. During the test phase, the animals did not receive any noxious stimulation or optogenetic treatment and had free access to both compartments for a total of 10 min. Animal movements in each of the chambers were recorded, and the time spent in either of the treatment chambers was analyzed by ANY-maze software. A preference score was calculated by subtracting the amount of time a rat stayed in the chamber associated with optogenetic activation during the test phase by the amount of time it stayed in that chamber at baseline.

Prior to behavioral tests, optic fibers were connected to a 589 nm laser through an FC/PC adapter (Shanghai Dream Lasers, Shanghai). The laser output intensity was measured with a power meter (Thorlabs, Newton, NJ, USA) prior to each experiment. The laser intensity was set at approximately 6 mW. Laser was delivered using a TTL pulse-generating box (Tucker-Davis Technologies, Alachua, FL, USA). The laser was turned on continuously during each trial of the cold allodynia and Hargreaves’ tests. It was turned on during the pairing of corticostriatal inhibition and noxious stimulations for the CPP and the conditioned place aversion (CPA) tests.

### Statistics

The results of behavioral experiments were given as mean ± SEM. For mechanical and cold allodynia, a two-way analysis of variance (ANOVA) with repeated measures and *post hoc* multiple pair-wise comparison Bonferroni tests were used to compare the 50% withdrawal threshold and cold score for SNI- treated and control rats. A two-tail paired Student’s *t-test* was used to analyze the results from the Hargreaves’ test. For CPP tests in SNI treated rats, differences in time spent in each chamber before conditioning (pre-conditioning) and after conditioning (test) were analyzed using a two-way ANOVA with repeated measures, followed by *post hoc* Bonferroni tests. A paired Student’s *t*-test was used to compare the time spent in each treatment chamber before and after conditioning (i.e., baseline vs. test phase for each chamber). Decreased time spent in a chamber during the test phase as compared with the baseline, indicates avoidance (aversion) for that chamber. A CPA score was computed by subtracting the time spent in the more noxious chamber during the test phase from the time spent in that chamber at baseline. A two-tailed unpaired Student’s *t-test* was used to compare differences in CPA scores under various testing conditions. For all tests, a *p* value <0.05 was considered statistically significant. All data were analyzed using GraphPad Prism Version 7 software (GraphPad, La Jolla, CA, USA).

## Results

### Inhibition of the PFC Pyramidal Neurons Enhances Nocifensive Reflex and Affective Response to Acute Pain

**Figure 1:**
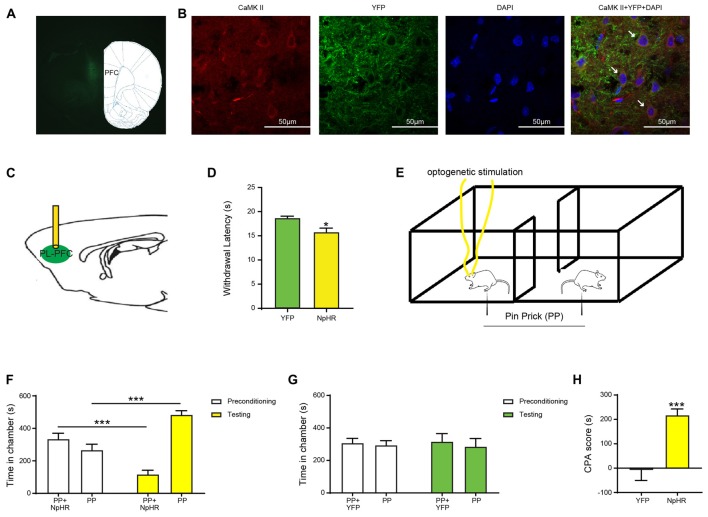
Inhibition of prefrontal cortex (PFC) pyramidal neurons enhances nocifensive reflex and affective response to acute pain.** (A)** Histologic expression of halorhodopsin in the prelimbic region of the PFC with low magnification (10×). **(B)** High-magnification (63×) view of the expression of halorhodopsin (NpHR)-YFP in the PL-PFC. From left to right: CaMK II staining; NpHR-eYFP staining; DAPI staining and merged images. **(C)** Schematic of optogenetic manipulation of the PL-PFC. **(D)** Optogenetic inhibition of the PL-PFC decreased the withdrawal latency in NpHR-treated rats. *n* = 8–11; *p* = 0.0112, unpaired Student’s *t*-test. **(E)** Schematic of the conditioned place aversion (CPA) test with optogenetic stimulation of the PL-PFC. Optogenetic stimulation was paired with PP in one chamber during conditioning phase, the other chamber was paired with PP. **(F,G)** Inhibition of PL-PFC increased the aversive response to acute pain. NpHR-treated rats displayed avoidance for the chamber associated with light stimulation. *n* = 7; *p* =0.0001, paired Student’s *t*-test. YFP-treated rats showed no preference for either chamber. *n* = 7; *p* = 0.8517, paired Student’s *t*-test. **(H)** Inactivation of PL-PFC enhanced the aversion to painful stimulation, as demonstrated by the increasing CPA score. *n* = 7; *p* = 0.0006, unpaired Student’s *t*-test. *p < 0.05, ***p < 0.001.

We expressed halorhodopsin (NpHR) in the pyramidal neurons of the PL-PFC, and then shone light to specifically inhibit these neurons during pain behavior tests (Figures 1A–C). First, we performed Hargreaves’ test to assess the impact of the PL-PFC in acute thermal pain regulation. We found that inactivation of the PL-PFC substantially decreased latency to withdrawal to the thermal stimulus, suggesting that this region produces endogenous nociceptive control (Figure 1D).

Pain has both sensory and affective components. To assess the affective component of pain, we used a well-known two-chamber conditioned place aversion assay (Johansen et al., 2001; King et al., 2009; Martinez et al., 2017; Zhang et al., 2017). During the preconditioning phase of this test, rats were allowed free access to both chambers. During the conditioning phase, one of the chambers was paired with repeated noxious mechanical stimulations in the form of a pin prick (PP) to the hind paw, coupled with simultaneous optogenetic inactivation of the PL-PFC. In contrast, the opposite chamber was paired with only PP. Finally, during the test phase, rats were allowed free access to both chambers again, without peripheral stimulations or optogenetic modulations (Figure 1E). We found that even though rats received peripheral noxious stimulations in both chambers, during the test phase they clearly avoided the chamber that was associated with PFC inhibition (Figure 1F). In contrast, YFP-expressing rats did not display avoidance of either chamber (Figure 1G). The aversive response to pain can be further quantified by a CPA score, which is calculated by subtracting the time rats stayed in the light chamber during the test phase from the preconditioning phase (Johansen and Fields, 2004; Zhang et al., 2017). When we compared rats that expressed NpHR and hence received PFC inhibition with control rats that received YFP expression, we found that NpHR rats demonstrated elevated CPA scores (Figure 1H). These results indicate that inhibiting the PFC, during the presentation of a noxious stimulus, increases the aversive value of that stimulus. Thus, PFC likely provides endogenous inhibition for the aversive, or affective, response to acute pain.

### Selective Inhibition of the Projection From the PL-PFC to the NAc Core Increases Sensory and Affective Responses to Noxious Stimulations

**Figure 2:**
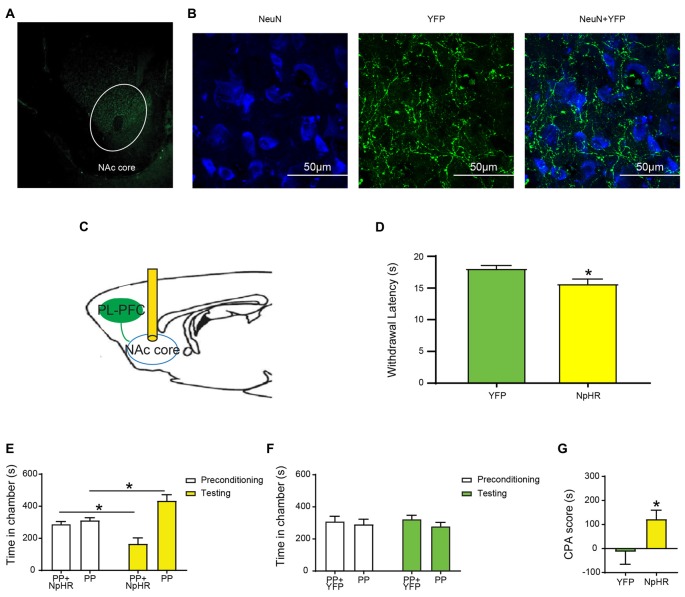
Selective inhibition of the projection from the PL-PFC to the Nucleus accumbens (NAc) core increases acute pain-related behaviors.** (A)** Low magnification (10×) view of NpHR-eYFP in the NAc core. **(B)** High magnification (100×) view of NpHR-eYFP in the NAc core. From left to right: NeuN staining; NpHR-eYFP staining; and merged images. **(C)** Schematic of inhibition of the PFC-NAc projection. Optic fibers were implanted bilaterally in the NAc core after injection of virus in the PL-PFC. Light treatment was delivered to the NAc core. **(D)** Optogenetic inactivation of the NAc core decreased the latency to paw withdrawal in Hargreaves’ test. *n* = 7–8; *p* = 0.0200, unpaired Student’s *t-test*. **(E)** Optogenetic inhibition of the PFC-NAc projection worsened the pain-related aversive response. *n* = 8; *p* = 0.0134, paired Student’s *t-test*. **(F)** YFP-treated rats demonstrated no preference to either chamber. *n* = 7; *p* = 0.8064, paired Student’s *t-test*. **(G)** Selective inhibition of the PFC-NAc core projection increased the aversive effect of painful stimulation, demonstrated by the increasing CPA score. *n* = 7–8; *p* = 0.0495, unpaired Student’s *t-test* *p < 0.05.

The PFC projects to the NAc to regulate reward-driven behaviors (Beckstead and Norgren, 1979; Sesack et al., 1989; Brog et al., 1993; Ishikawa et al., 2008). This projection has also been shown to be involved in either the processing or the regulation of pain (Baliki et al., 2012). Furthermore, recent studies indicate that supraphysiologic activations of the PL-PFC to NAc core projection can inhibit acute and chronic pain phenotypes (Lee et al., 2015; Martinez et al., 2017). However, it is not yet clear whether this circuit provides endogenous pain regulation. To address this question, we expressed NpHR in the pyramidal neurons of the PL-PFC, and then inserted optic fibers in the NAc core (Figures 2A–C). This approach allowed us to selectively inhibit the synaptic transmission between the axons of the PFC neurons and the dentrites of NAc neurons. We found that the inhibition of this synaptic projection shortened the latency to withdrawal on the Hargreaves’ test (Figure 2D). We then paired optogenetic inhibition of this corticostriatal projection with PP in one of the chambers during the conditioning phase of the CPA; the other chamber was paired with PP without optogenetic inhibition. During the test phase of the CPA, rats that expressed NpHR avoided the chamber paired with PP and corticostriatal inhibition (Figure 2E). In contrast, control (YFP) rats did not demonstrate this avoidance (Figure 2F). These results were further quantitated by the enhanced CPA score for rats that expressed NpHR compared with control rats (Figure 2G). Thus, inhibition of this corticostriatal projection enhances both the sensory and affective components of acute pain, suggesting a critical pain-inhibitory role for this circuit in naïve rats.

### Inhibition of the Corticostriatal Circuit Enhances Noxious Stimulus Triggered Behaviors in Rats With Chronic Pain

Previous work suggests that the projection from the PFC to the NAc can be altered in the chronic pain state (Geha et al., 2008; Baliki et al., 2012), and optogenetic activation of this pathway can inhibit the nocifensive reflex and the aversive response to chronic pain (Lee et al., 2015; Martinez et al., 2017). Thus, we investigated the role for this corticostriatal projection in the endogenous regulation of nociceptive response in a chronic pain model.

**Figure 3:**
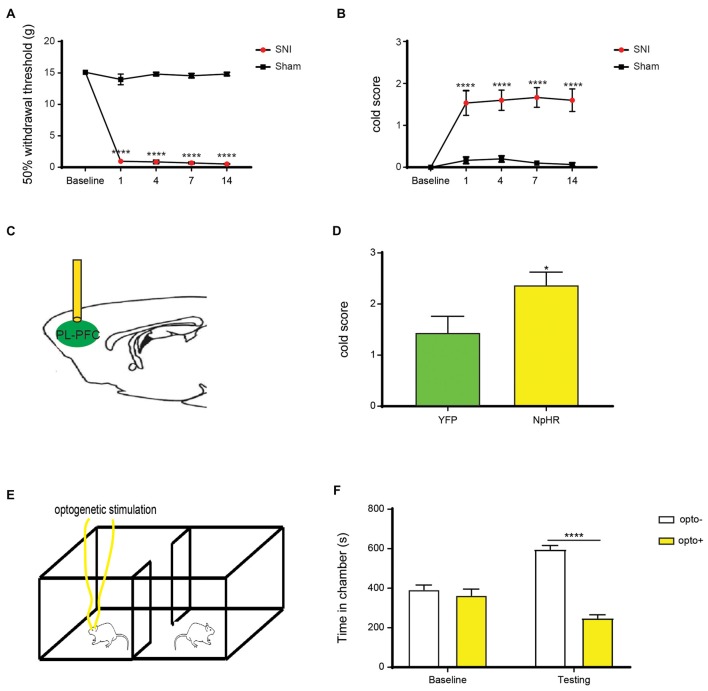
Inhibition of the PL-PFC enhances sensory allodynia and the affective response to pain in the chronic pain state. **(A,B)** Rats developed sensory impairment after spared nerve injury (SNI) surgery. Two-way analysis of variance (ANOVA) with repeated measures and Bonferroni’s post-test, *n* = 6, *p* < 0.0001. **(C)** Schematic of the light treatment in the PL-PFC. **(D)** Deactivation of the PL-PFC increased cold allodynia in rats with neuropathic pain. *n* = 6–8; *p* = 0.0389, unpaired Student’s *t-test*. **(E)** Schematic showing optogenetic deactivation of PL-PFC during the Conditioned Place Preference (CPP) testing for SNI-treated rats. Neither chamber was associated with peripheral stimulations, and rats were conditioned for 4 days to assess the aversive response to chronic pain. **(F)** CPP data shows that SNI-treated rats displayed a significant avoidance for the chamber paired with PFC inhibition. *n* = 6, *p* < 0.0001. Two-way ANOVA with repeated measures and Bonferroni post-test *p < 0.05,****p < 0.0001.

We used a well-known SNI procedure to induce chronic neuropathic pain in rats (Decosterd and Woolf, 2000; Goffer et al., 2013). We resected tibial and common peroneal branches of the sciatic nerves of rats, leaving the sural nerves intact. As expected, after this procedure, rats demonstrated persistent symptoms of mechanical and cold allodynia (Figures 3A,B). Next, we examined how the inhibition of the projection from the PFC to the NAc would impact sensory and affective phenotypes of chronic neuropathic pain. First, we inhibited pyramidal neurons of the PL-PFC (Figure 3C). We found that inhibiting the PFC increased cold allodynia, an index for sensory pain transmission (Figure 3D). To assess the impact of PFC inhibition on the affective symptoms of chronic pain, we performed a traditional multi-day CPP assay. In multiple rodent pain models, this assay has been used to investigate the negative reinforcement associated with the seeking of pain relief, as well as to unmask the aversive quality of ongoing or spontaneous pain (Johansen et al., 2001; King et al., 2009; Navratilova et al., 2012; Daou et al., 2013; De Felice et al., 2013). During the conditioning phase of this test, we paired PFC inactivation with one chamber, and no PFC modulation with the other chamber (Figure 3E). After 4 days of conditioning, during the test phase, SNI-treated rats avoided the chamber associated with PFC inhibition (Figure 3F). These results suggest that inhibition of the PFC output enhances the affective experience of chronic pain, particularly during spontaneous or tonic episodes of pain, in addition to its regulatory effect on sensory allodynia.

**Figure 4:**
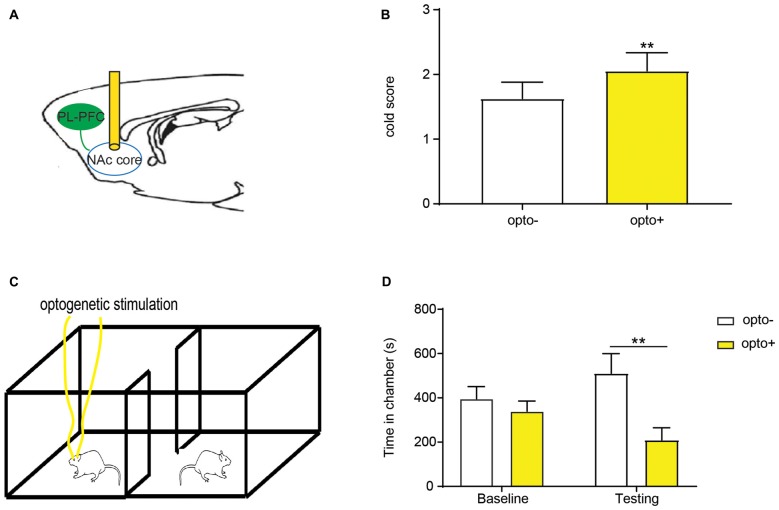
Deactivation of the PFC-NAc projection worsened both sensory and affective components of chronic pain. **(A)** Selective deactivation of the PFC-NAc circuit through light delivery into the NAc core. **(B)** Sensory symptoms were worsened with inhibition of the PFC-NAc projection in the chronic pain state. *n* = 7; *p* = 0.0082, paired Student’s *t-test*. **(C)** CPP test was performed in the SNI-treated rats with selective inhibition of the NAc core in one chamber, and no light stimulation in the other. **(D)** SNI-treated rats preferred the chamber associated without light stimulation in the CPP test. *n* = 5; *p* = 0.0078. Two-way ANOVA with repeated measures and Bonferroni post-test **p < 0.01.

Next, we specifically inhibited the projection from the PL-PFC to the NAc core by expressing NpHR in the PL-PFC, inserting optic fibers into the NAc and shining light through the fibers (Figure 4A). Inhibition of this corticostriatal projection increased cold allodynia (Figure 4B), suggesting a role of this circuit in endogenous inhibition of the nocifensive reflex. Next, we paired one of the chambers with the inactivation of this circuit, and the other chamber without inactivation, during the 4-day conditioning phase of the CPP in the SNI-treated rats (Figure 4C). During the test phase, NpHR-expressing rats demonstrated avoidance of the chamber associated with light treatment, indicating that the selective inhibition of the axonal projection from the PFC to the NAc increases the aversive response to chronic pain (Figure 4D). Together, these results indicate that the projection from the PFC to the NAc plays a key role in endogenous nociceptive inhibition in the chronic pain state.

### Inhibition of the Corticostriatal Circuit Produces Similar Phenotypes as Chronic Pain

Our results indicate that inactivation of the corticostriatal projection enhances chronic pain. Previous work has demonstrated that this projection may be altered in the chronic pain state (Geha et al., 2008; Baliki et al., 2012). Thus, an interesting hypothesis is that impairment of the corticostriatal projection can contribute to the phenotype of chronic pain. To assess this possibility, we examined the impact of corticostriatal inhibition on a unique phenotype associated with chronic pain—generalized enhancement of aversion. Chronic pain patients are known to demonstrate increased aversive responses to acute pain in an anatomically non-specific manner (Scudds et al., 1987; Petzke et al., 2003; Kehlet et al., 2006; Kudel et al., 2007; Scott et al., 2010). For example, patients with fibromyalgia show increased emotional reactions to peripheral noxious stimuli in a diffuse, whole body-wide distribution. This anatomically nonspecific, or generalized, amplification of pain aversion, represents a key affective symptom of chronic pain. A recent study has also shown this phenotype in a rodent inflammatory pain model (Zhang et al., 2017). Hence, we investigated the role that the PFC-NAc circuit plays in this important chronic pain phenotype.

**Figure 5:**
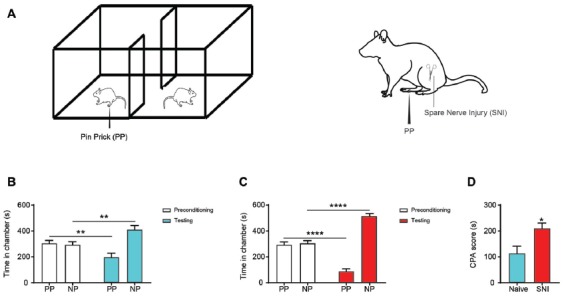
Chronic pain induced generalized enhancement of aversive response to peripheral nociceptive inputs. **(A)** CPA tests were performed with a PP to the uninjured paw or the paw contralateral to surgery. One chamber was paired with PP, and the other was not paired with painful stimulation (NP). **(B)** Rats that underwent sham surgery presented preference to chamber paired without acute pain. *n* = 10, *p* = 0.0046. Paired Student’s *t*-test. **(C)** SNI-treated rats displayed increased an aversive response to acute pain. *n* = 10, *p* < 0.0001. Paired Student’s *t-test*. **(D)** Chronic pain induced an enhanced pain-related aversion, demonstrated by increasing CPA score. *n* = 10, *p* = 0.0170. Unpaired Student’s *t-test* *p < 0.05, **p < 0.01, ****p < 0.0001.

First, to understand whether chronic neuropathic pain can induce a similar site-nonspecific enhancement in the aversive response to noxious stimulations, we conducted CPA assays by stimulating the opposite, non-injured paws with PP during conditioning in SNI-treated rats (Figure 5A). Thus, during conditioning, one of the chambers was paired with PP, and the other chamber was not paired with noxious stimulations (NP). As expected, during the test phase, control rats that did not experience chronic neuropathic pain, avoided the chamber associated with PP, demonstrating appropriate aversive responses (Figure 5B). SNI-treated rats, however, showed a substantial increase in their aversive response to noxious stimulation of the uninjured foot (Figure 5C), compared with control rats (Figure 5D). These results demonstrate that rats with chronic neuropathic pain display increased aversive responses to acute pain in an anatomically nonspecific manner, compatible with previous results from other chronic pain models (Zhang et al., 2017).

**Figure 6:**
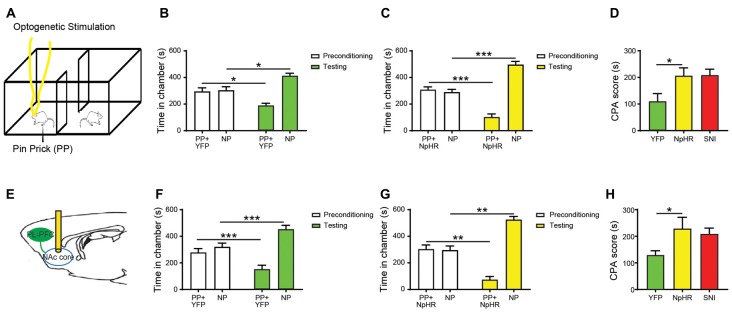
Inhibition of the corticostriatal circuit produces similar phenotypes as chronic pain.** (A)** Optogenetic CPA assays were performed in the rats without chronic pain. Light treatment was paired with PP in one chamber during the conditioning phase, the other chamber was paired without peripheral or optogenetic stimulation. **(B)** YFP-treated rats showed avoidance for the chamber with acute pain and light. *n* = 7, *p* = 0.0116. Paired Student’s *t-test*. **(C)** Inhibition of PL-PFC induced the enhanced aversive response to nociceptive inputs. *n* = 7, *p* = 0.0004. Paired Student’s *t-test*. **(D)** The CPA score demonstrated that the inhibition of PL-PFC increased the aversive effect of acute pain in rats without chronic pain. *n* = 7, *p* = 0.0399. Unpaired Student’s *t-test*. **(E)** Inhibition of the PFC-NAc projection was paired with PP during conditioning phase in the CPA testing. **(F)** YFP-treated rats developed avoidance for the chamber conditioned with PP and light treatment. *n* = 7, *p* = 0.0002. Paired Student’s *t-test*. **(G)** NpHR-treated rats displayed significant avoidance to the chamber paired with light and PP. *n* = 7, *p* = 0.0016. Paired Student’s *t-test*. **(H)** Inactivation of the PFC-NAc projection produced a similar aversive response to acute pain as to chronic pain, as demonstrated by the increasing CPA score. *n* = 7, *p* = 0.0463. Unpaired Student’s *t-test* *p < 0.05, **p < 0.01, ***p < 0.001.

Next, we compared the effect of inhibition of the PFC-NAc projection with the effect of chronic pain on the aversive response to noxious stimulations. We paired one chamber with PL-PFC inactivation and PP, and the other chamber without either cortical inhibition or peripheral stimulation (Figure 6A). Control (YFP-expressing) rats demonstrated appropriate avoidance of the PP chamber (Figure 6B). NpHR-expressing rats, however, displayed an increased avoidance of the PP chamber (Figure 6C), and showed an increased CPA score (Figure 6D). We then paired one chamber with selective inactivation of the PFC-NAc projection and PP, and the other chamber with NP (Figure 6E). Similar to what we found with PFC inactivation, specific inhibition of the corticostriatal pathway resulted in an increased aversive response to the noxious stimulus (Figures 6F–H). Finally, we compared the CPA scores for noxious stimulations under the condition of corticostriatal inhibition in naïve rats with CPA scores for noxious stimulations in rats with chronic pain. We found that these CPA scores are nearly identical, suggesting that diminished strength of this projection likely plays a role in the affective phenotype of chronic pain (Figure 6H).

## Discussion

The PFC provides top-down regulation of sensory and affective processes. Its projections to the PAG, thalamus and amygdala have been shown to impact chronic pain phenotypes (Bushnell et al., 2013; Cardoso-Cruz et al., 2013; Ji and Neugebauer, 2014). Recently, the projection from the PL-PFC to the NAc core, an important pathway within the reward circuitry (Koob and Volkow, 2010), has been activated to inhibit both acute and chronic pain behaviors in rodents (Lee et al., 2015; Zhang et al., 2015; Martinez et al., 2017). While these stimulation experiments indicate the capability of this corticostriatal circuit to exert pain control, no studies have since demonstrated if this pathway is indeed engaged in endogenous pain regulation. Furthermore, it is not known whether the impairment in this projection is responsible for chronic pain phenotypes. Our results demonstrate that the inhibition of this pathway makes pain worse, thereby strongly indicating that this pathway is employed endogenously to suppress pain. Furthermore, we show that the inactivation of this circuit in naïve rats can produce the aversive symptoms of chronic pain.

Our results here show that inhibition of the PFC-NAc projection has exactly the opposite effect as the stimulation of this corticostriatal pathway, as demonstrated in earlier investigations (Lee et al., 2015; Martinez et al., 2017). Together, results from these studies indicate that this corticostriatal circuit can provide bidirectional regulation of pain phenotypes. Excitation of this circuit inhibits these phenotypes, whereas its inactivation enhances them. Furthermore, what is notable is that this projection can regulate both sensory and affective components of pain. The sensory pain control is manifested by changes in the withdrawal latency on Hargreaves’ test of acute thermal nociception, and in cold allodynia in the SNI model. These changes in nocifensive withdrawal behaviors indicate that the PFC-NAc projection can influence the nociceptive transmission at the spinal level. The PAG-RVM circuit is a well-known conduit for descending pain regulation (Fields et al., 1983; Morgan et al., 1989), and there is evidence that the NAc can project to neurons in these two brain regions (Yu and Han, 1990; Gear et al., 1999; Becerra et al., 2001; Magnusson and Martin, 2002; Becerra and Borsook, 2008; Geha et al., 2008; Gear and Levine, 2009; Baliki et al., 2010; Goffer et al., 2013). Therefore, the PFC-NAc circuit may function upstream from the PAG-RVM pathway in nociceptive regulation.

In addition to its modulation of sensory pain pathways, both the PFC and the NAc have been shown to regulate mood and affect. The PFC is well-studied for its role in top-down control of emotion. The NAc, meanwhile, has recently emerged as a key brain area for mood regulation based on studies of depression and addiction (Park et al., 2005; Berton et al., 2006; Nestler and Carlezon, 2006; Lim et al., 2012; Golden et al., 2013). Furthermore, activation of the NAc has been implicated in the negative reinforcement of pain relief (De Felice et al., 2013; Xie et al., 2014). The NAc can project to the ventral pallidum and substantia nigra (Nestler and Carlezon, 2006), and neurons from these regions in turn terminate in the ventral anterior, dorsal and lateral thalamus, which project to the PFC and anterior cingulate cortex (ACC). Thus, the NAc and the PFC may be a part of a larger, multi-component, striato-thalamo-cortical loop. Previous studies have delineated the function of this network in mood regulation (Russo and Nestler, 2013). Furthermore, the interaction between ACC and NAc has also been suggested to play a role in the aversive processing of chronic pain (De Felice et al., 2013; Xie et al., 2014). Thus, our results are compatible with these previous studies and suggest that this cortico-subcortical network is endogenously involved to provide the affective evaluation and response to pain.

A key finding in our study is that inactivation of the PFC-NAc projection captured a very important aversive phenotype of chronic pain. Patients who suffer from fibromyalgia or persistent postoperative pain, are known to demonstrate an increased aversive response to noxious stimulations in an anatomically non-specific manner (Scudds et al., 1987; Petzke et al., 2003; Kehlet et al., 2006; Kudel et al., 2007; Scott et al., 2010). This enhanced emotional reaction to peripheral noxious stimuli, in a diffuse, whole body-wide distribution, can contribute to meaningful clinical symptoms, including magnified pain anticipation and pain catastrophization (Meints and Edwards, 2018). In this study we confirmed that this anatomically nonspecific, or generalized, amplification of pain aversion can also be demonstrated in rodent chronic pain models (Zhang et al., 2017). When we compared corticostriatal inhibition with chronic pain, we found that CPA scores for these conditions were nearly identical, suggesting that the inhibition of this projection could play a role in this chronic pain phenotype. Interestingly, studies in chronic pain patients have demonstrated a decrease in prefrontal gray matter, as well as altered prefrontal projections to other subcortical regions (Apkarian et al., 2004; Geha et al., 2008; Moayedi et al., 2011). In addition, investigations in animal chronic pain models have shown that impaired glutamate, endocannabinoid and cholinergic signaling all contribute to decreased prefrontal outputs (Ji and Neugebauer, 2011; Zhang et al., 2015; Kelly et al., 2016; Radzicki et al., 2017). Together, these data suggest diminished prefrontal outputs to regions such as the NAc in the chronic pain state. Putting our work in this conceptual framework, decreased PFC projections to the NAc may play a significant role for the aversive phenotype of chronic pain.

The PFC is a structurally and functionally heterogenous region. In rodents, it can be subdivided into three components: the ACC, PL and infralimbic (IL) regions. Our work is based on the projection from the PL to the NAc core subregion. While the IL has also been shown to possess pain-relieving properties (Kiritoshi et al., 2016), it provides only weak projections to the shell subregion of the NAc (Beckstead and Norgren, 1979; Sesack et al., 1989; Sesack and Pickel, 1992; Brog et al., 1993; Vertes, 2004). Meanwhile, inhibition of the ACC, a major source of inputs to the NAC, has been shown to decrease the aversive response to noxious stimulations, and its stimulation has opposite effects (Johansen and Fields, 2004; Zhang et al., 2017). Interestingly, these components of the PFC have been shown to be capable of mutual inhibition or excitation (Ji and Neugebauer, 2012; Riga et al., 2014). Therefore, dynamic interactions of different subregions of the PFC can give rise to a diverse set of functions, and as the result of this cortical dynamic, the PFC can, through its outputs to the NAc, achieve endogenous regulation of both sensory and aversive components of pain. Chronic pain, meanwhile, may have the capacity to alter such cortico-cortical and cortico-subcortical dynamics. Future studies are needed to fully dissect this complex functional pain circuitry.

In addition to aversion, pain can produce a host of emotional responses, including depression and anxiety (Romano and Turner, 1985; Dworkin and Gitlin, 1991; Ohayon and Schatzberg, 2003; Edwards et al., 2009; Miller and Cano, 2009; Scott et al., 2010). Previous studies in animal models of acute and chronic pain have validated such findings, and have identified the involvement of both the PFC and the NAc in such affective phenotypes (Wang et al., 2011; Goffer et al., 2013; Stratinaki et al., 2013; Schwartz et al., 2014; Barthas et al., 2015; Mitsi et al., 2015; Sellmeijer et al., 2018). Our previous study has indicated that activation of the projection from the PL-PFC to the NAc core, can inhibit the depressive phenotype triggered by chronic pain (Lee et al., 2015). Based on our results here, it is likely that the inhibition of this pathway will enhance the phenotypes of depression and anxiety associated with pain. Future studies shall determine the exact involvement of this circuit in the endogenous regulation of mood in pain states.

In our study, optogenetic silencing of the corticostriatal circuit inhibited pain behaviors, as manifested by changes in the spinal withdrawal reflex and aversive response. An alternative explanation of our data is that such behavioral changes are the results of a complex interaction between acute or chronic pain and cortico-subcortical modulation. While we cannot completely rule out such a complex interaction, our results on the comparison in the CPA scores between corticostriatal silencing and SNI treatment (Figure 6) indicate that corticostriatal inhibition likely provides independent pain-relieving effects. However, future studies of *in vivo* physiology may be suited to fully dissect such interaction.

In summary, in the current study we addressed an important question in sensory neuroscience: does the cortex project to the striatum to control pain endogenously? To answer this question, we inhibited the projection from the PL-PFC to the NAc core in rats. We found that the inhibition of this pathway enhanced both acute and chronic pain phenotypes, furthermore, inhibition of this corticostriatal projection had similar effects on aversive processing as chronic pain. These results indicate that the projection from the PFC to the NAc plays an important role in the endogenous regulation of pain, and impairment in this circuit can contribute to the pathology of chronic pain. These results also suggest that neurostimulation strategies that can specifically target this endogenous pain regulatory pathway may constitute a novel therapeutic option.

## Author Contributions

DH and JW designed the project. HZ, RY and JAD performed viral injections and optic fiber implantations. HZ and EM performed immunohistological staining. HZ, EM, HL, RY, JAD and KL performed behavioral tests and data analysis. JW wrote the article.

## Conflict of Interest Statement

The authors declare that the research was conducted in the absence of any commercial or financial relationships that could be construed as a potential conflict of interest.
